# WWOX dysfunction induces sequential aggregation of TRAPPC6AΔ, TIAF1, tau and amyloid *β*, and causes apoptosis

**DOI:** 10.1038/cddiscovery.2015.3

**Published:** 2015-08-03

**Authors:** J-Y Chang, N-S Chang

**Affiliations:** 1 Institute of Molecular Medicine, National Cheng Kung University Medical College, Tainan, Taiwan; 2 Center for Infectious Disease and Signaling Research, National Cheng Kung University Medical College, Tainan, Taiwan; 3 Advanced Optoelectronic Technology Center, National Cheng Kung University Medical College, Tainan, Taiwan; 4 Department of Neurochemistry, New York State Institute for Basic Research in Developmental Disabilities, Staten Island, NY, USA

## Abstract

Aggregated vesicle-trafficking protein isoform TRAPPC6AΔ (TPC6AΔ) has a critical role in causing caspase activation, tau aggregation and A*β* generation in the brains of nondemented middle-aged humans, patients with Alzheimer’s disease (AD) and 3-week-old *Wwox* gene knockout mice. WWOX blocks neurodegeneration via interactions with tau and tau-phosphorylating enzymes. WWOX deficiency leads to epilepsy, mental retardation and early death. Here, we demonstrated that TGF-*β*1 induces shuttling of endogenous wild-type TPC6A and TPC6AΔ in between nucleoli and mitochondria (~40–60 min per round trip), and WWOX reduces the shuttling time by 50%. TGF-*β*1 initially maximizes the binding of TPC6AΔ to the C-terminal tail of WWOX, followed by dissociation. TPC6AΔ then undergoes aggregation, together with TIAF1 (TGF-*β*1-induced antiapoptotic factor), in the mitochondria to induce apoptosis. An additional rescue scenario is that TGF-*β*1 induces Tyr33 phosphorylation and unfolding of WWOX and its the N-terminal WW domain slowly binds TPC6AΔ to block aggregation and apoptosis. Similarly, loss of WWOX induces TPC6AΔ polymerization first, then aggregation of TIAF1, amyloid *β* and tau, and subsequent cell death, suggesting that a cascade of protein aggregation leads to neurodegeneration.

## Introduction

In a recent study, we reported the isolation of a truncated vesicle-trafficking protein TRAPPC6AΔ (TPC6AΔ).^1^ Wild-type TRAPPC6A (TPC6A) is one of the components in the transport protein particle (TRAPP) complex.^2–4^ *TRAPPC6A* gene has been implicated in the neurodegenerative disease.^5^ TPC6AΔ has an internal deletion of 14 amino acids at the N terminus. Unlike the wild-type protein, TPC6AΔ readily undergoes aggregation in the extracellular matrix.^1^ TPC6AΔ aggregates or plaques have been found in the brain cortex and hippocampus in nondemented middle-aged humans and in patients with Alzheimer’s disease (AD), suggesting that TPC6AΔ is a marker for early onset of AD.^1^ Also, in 3-week-old *Wwox* gene knockout mice,^1^ aggregates of TPC6AΔ, TIAF1 (TGF-*β*-induced antiapoptotic factor),^6,7^ tau and amyloid beta (A*β*) can be found in the brain cortex. We have determined that downregulation of WWOX leads to the aggregation of TPC6AΔ, TIAF1, tau and A*β in vitro* and *in vivo.*^1,7–9^ Sequentially, upon WWOX downregulation, TPC6AΔ becomes aggregated first, followed by TIAF1 aggregation, caspase activation, APP degradation for A*β* generation, as well as tau tangles formation.^1,7–9^ The observations suggest a critical role of WWOX in regulating protein aggregation and AD progression.

Human and mouse *WWOX/Wwox* gene, which encodes tumor suppressor WW domain-containing oxidoreductase (known as WWOX, FOR or WOX1), is known to have a critical role in blocking neurodegeneration.^9–13^ WWOX possesses two N-terminal WW domains, a C-terminal short chain alcohol dehydrogenase/reductase domain (SDR) and a nuclear localization signal in between the domains.^10,11,14–18^ WWOX is essential for embryonic neural development.^9,19^ However, under stress conditions (e.g., axotomy and constant light), there is an increased expression of WWOX, along with Tyr33 phosphorylation, that allows the activated protein to undergo nuclear accumulation and cause neuronal injury and damage.^20–22^ When activated WWOX is localized in the nucleus, its N-terminal WW domain may induce the transcription activation of NF-*κ*B-dependent promoter.^22^ Whether this contributes, in part, to axotomy-induced acute and chronic brain inflammation is unknown and remains to be established.^22^

We have determined that WWOX is significantly downregulated in the AD hippocampus,^8^ indicating that loss of WWOX promotes AD progression. Most recently, important studies showed that homozygous nonsense mutations or other alterations of *WWOX* gene result in protein loss and cause patients to suffer from severe anomalies, including short stature and growth retardation, microcephaly with seizure, retinal degeneration and early death at 16 months of age.^13,23–27^ We have determined that WWOX blocks neurodegeneration via binding tau and tau-phosphorylating enzymes, including ERK, JNK and GSK-3*β*.^8,28,29^ Also, WWOX stimulates neuronal differentiation.^29^ Together, WWOX possesses dual functions in controlling cell death or survival. Under physiological conditions, WWOX exerts its homeostatic role by using WW domain for signaling and the SDR domain for metabolism. Without WWOX, cells tend to undergo rapid proliferation and apoptosis. Under stress conditions, increased levels of pY33-WWOX are accumulated in the mitochondria and nuclei for causing cell death.

Here, we show that in response to transforming growth factor beta (TGF-*β*), both wild-type TPC6A and TPC6AΔ shuttle from nucleus, nucleolus and then to mitochondrion, and again travel back to the nucleus. The nucleolus–mitochondrion shuttling is novel. However, when TPC6AΔ undergoes excessive aggregation in the mitochondria, activation of caspases occurs that leads to apoptosis.^1,7,30^ TGF-*β*/Smad signaling has a critical role in the pathogenesis of AD.^7,31,32^ TGF-*β*1 expression is upregulated in a transgenic mouse model of familial Alzheimer's disease, and this leads to neuronal apoptosis.^32^ How WWOX interacts with TPC6A under the effect of TGF-*β*1 was examined by co-immunoprecipitation (co-IP) and time-lapse microscopy with Förster (fluorescence) resonance energy transfer (FRET) analysis.^22,28,33,34^

## Results

### Polymerization of endogenous TPC6A in the nuclei and mitochondria

**Figure 1:**
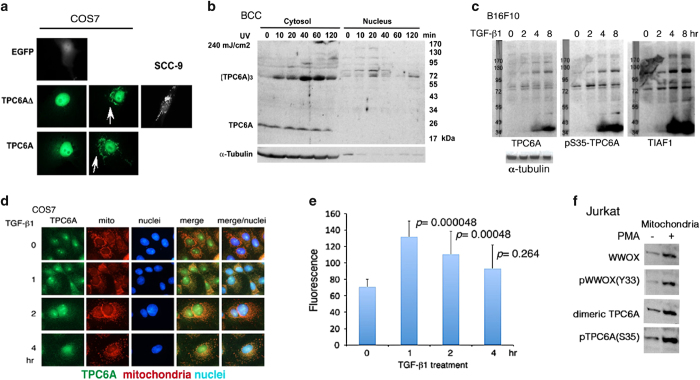
TPC6A protein aggregation in subcellular compartment. (**a**) Ectopic expression of wild-type TPC6A and an isoform TPC6AΔ in COS7 and SCC-9 cells results in localization of the proteins in the nucleus and cytoplasm. Perinuclear protein aggregation is shown in most cases (see white arrows). Control cells expressing EGFP do not exhibit protein aggregation. (**b**) Basal cell carcinoma (BCC) cells were exposed to UV light, followed by incubation and then harvesting at indicated times. UV irradiation quickly induced formation of endogenous trimeric TPC6A in both cytosol and nucleus in 10–20 min. A representative data from two experiments is shown. (**c**) B16F10 melanoma cells were treated with TGF-*β*1 (5 ng/ml) for various durations. Presence of aggregates of TIAF1 and TPC6A and its Ser35 phosphorylation is shown (non-reducing SDS-PAGE). No ubiquitin attachment to these proteins was observed (data not shown). Protein size markers are on the left column. Monomeric TIAF1 is 12 kDa and TPC6A is 17–20 kDa. A representative data from two experiments is shown. (**d** and **e**) COS7 cells were treated with TGF-*β*1 (5 ng/ml) for indicated durations and stained with a specific antibody against TPC6A and MitoTracker Red for mitochondria, respectively. Confocal microscopy analysis revealed that TGF-*β*1 induced TPC6A relocation to the mitochondria, as revealed by increased yellow fluorescence in co-localization analysis. (**f**) PMA at 10 *μ*M stimulated relocation of TPC6A and WWOX, along with their phosphorylated forms, to the mitochondria in Jurkat T cells during the treatment for 90 min.

We have recently demonstrated that downregulation of WWOX leads to aggregation of wild-type TPC6A and TPC6AΔ both in *Wwox* knockout mice and in knockdown experiments using cell lines.^1^ Compared with the wild type, TPC6AΔ can readily undergo aggregation in the extracellular matrix of the brain.^1^ To further validate whether polymerization or aggregation of TPC6AΔ is universal, different types of mammalian cell lines were used. Transient overexpression of EGFP-TPC6A or -TPC6AΔ in COS7 fibroblasts by electroporation resulted in localization of both the proteins mainly in the nucleus (Figure 1a). Cytosolic aggregates of EGFP-TPC6A and -TPC6AΔ proteins are also shown (Figure 1a; see white arrows). Aggregate formation was also observed by overexpressing TPC6AΔ in cutaneous squamous cell carcinoma SCC-9 cells (Figure 1a) and many cell types (data not shown).

In cutaneous basal cell carcinoma BCC cells, endogenous TPC6A is a 20-kDa monomer in the cytoplasm, and becomes a trimer or larger sizes in the nuclei (~70 kDa and larger), as determined by western blotting (Figure 1b). Exposure of BCC cells to UV irradiation rapidly induced the formation of a trimeric form in 10 min and aggregates of >200 kDa in 60–120 min in the cytoplasm (Figure 1b). TPC6A appeared to relocate to the nucleus and became aggregated in 20 min post UV exposure, followed by disappearance from the nucleus (Figure 1b). The aggregates appear to be metabolically degradable, whereas no ubiquitination was shown with these proteins (data not shown). Similarly, transforming growth factor beta 1 (TGF-*β*1) induced self-aggregation of TPC6A and Ser35-phosphorylated TPC6A, and the aggregation exhibited in a ladder-like pattern in melanoma B16F10 cells (Figure 1c). In agreement with our previous observations,^7^ TGF-*β*1 caused TIAF1 aggregation (Figure 1c).

The endogenous TPC6A is abundant in the nuclei and nucleoli of COS7 cells (Figure 1d). Confocal microscopy analysis revealed that TPC6A was accumulated in the nucleoli and nuclei, and stimulation of these cells with TGF-*β*1 resulted in the relocation of TPC6A to the mitochondria, as determined by co-localization analysis (Figures 1d and e). We reported that phorbol 12-myristate 13-acetate (PMA) induces the relocation of WWOX to the mitochondria in Jurkat T cells.^28^ When Jurkat T cells were stimulated with PMA (5 *μ*M) for 90 min, endogenous TPC6A and WWOX, along with their phosphorylated forms, were shown in the mitochondria (Figure 1f). TPC6A was present mainly as a monomer in the cytoplasm (data not shown), whereas it became a dimer in the mitochondria (Figure 1f). In other cell types (e.g., monocytic THP-1 and U937 cells), mitochondrial TPC6A may exist as a trimer (data not shown). In addition, in response to TGF-*β*1, we have demonstrated the relocation of WWOX, along with p53, to the mitochondria and nuclei.^15,28,35^

### TGF-*β*1 induces endogenous TPC6A to shuttle in between nucleoli and mitochondria

**Figure 2:**
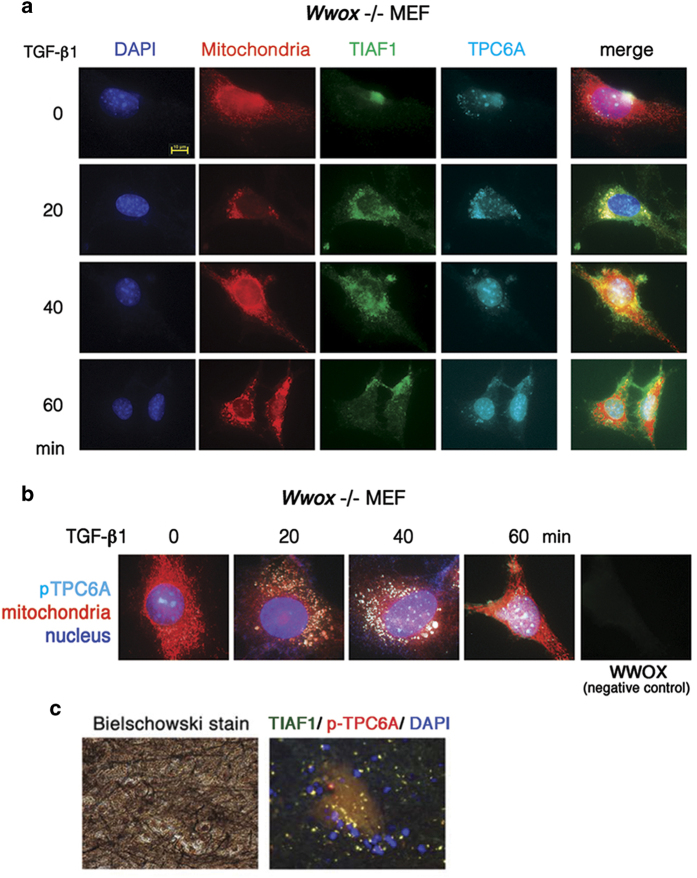
TGF-*β*1 induces a two-way shuttling of endogenous TPC6A in between nucleoli and mitochondria. (**a**) *Wwox*−/− MEF cells were exposed to TGF-*β*1 (5 ng/ml), and then stained with specific antibodies for TPC6A and TIAF1. Mitochondria were stained with MitoTracker Red. Wild-type TPC6A shuttles in between nucleoli to mitochondria. Each round trip takes ~60 min. Endogenous TIAF1 is mainly retained in the mitochondria. Approximately 100–120 cells were examined. (**b**) Similar results were observed by staining Ser35-phosphorylated TPC6A with a specific antibody. (**c**) TIAF1 co-localizes with TPC6A with Ser35 phosphorylation in the extracellular matrix as aggregates in the human AD hippocampus (also see Supplementary Figure S1).

We have established *Wwox*−/− MEF cells.^1^ These cells were exposed to TGF-*β*1, followed by determining the localization of TPC6A. TGF-*β*1 induced translocation of the wild-type TPC6A from the nucleoli to the mitochondria in 20–30 min. TPC6A then traveled back to the nucleoli in about the same duration (Figures 2a and b). Together, a round trip of TPC6A shuttling in between mitochondria and nucleoli is around 40–60 min. In the wild-type *Wwox*+/+ MEF cells, the round trip of shuttling time for TPC6A drops down to 20 min, suggesting that WWOX increases the speed of TPC6A shuttling. Indeed, similar results were observed in WWOX-expressing COS7 cells. The round trip of shuttling time for TPC6A is ~10 min in COS7 cells. Endogenous TIAF1 was mainly retained in the mitochondria in *Wwox*−/− MEF cells (Figure 2a). In the AD hippocampus, TIAF1 co-localizes with Ser35-phosphorylated TPC6A in the extracellular matrix as aggregates (Figure 2c and Supplementary Figure S1). We have determined that TIAF1 participates in the TGF-*β*1 signaling via binding with Smad4, and that Smad4 prevents self-aggregation of TIAF1.^6,7,36^ Transiently overexpressed TIAF1 acts together with p53 and WWOX in inducing apoptosis.^30^

### TPC6A physically binds to the C-terminal D3 tail of WWOX

**Figure 3:**
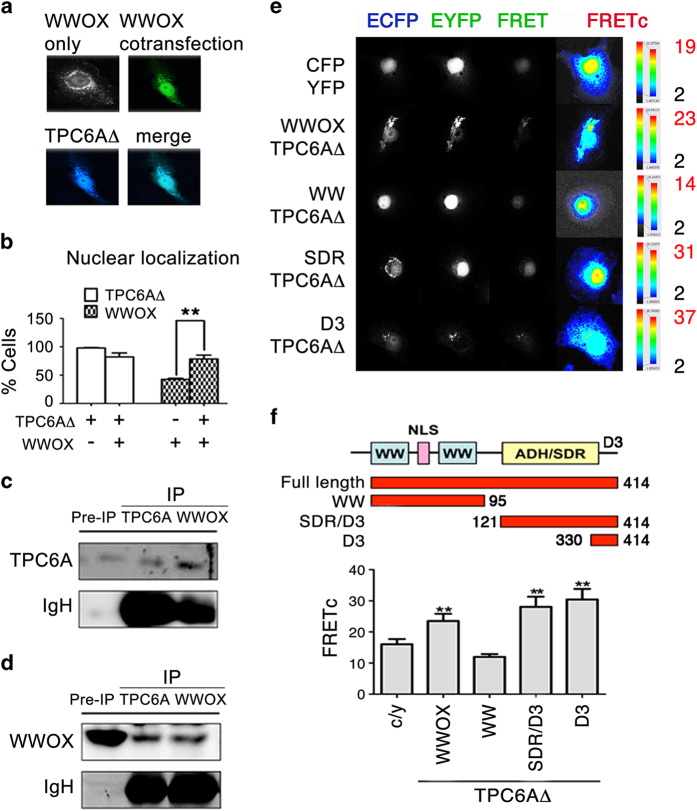
TPC6A physically binds the C-terminal D3 tail of WWOX. (**a** and **b**) Transiently overexpresssed EGFP-WWOX localized mainly in the perinuclear area in COS7 cells. Co-expression of WWOX and TPC6AΔ results in increased nuclear accumulation of WWOX compared with WWOX expression alone (~100 cells counted, mean±S.D., *n*=3; **P<0.05, Student’s *t*-test). (**c** and **d**) HEK293 cells were cultured in 10% FBS/medium and grown in a 10-cm dish to 100% confluence. Cell lysates were prepared and processed for co-immunoprecipitation using specific antibodies, respectively, against TPC6A and WWOX. Western blotting analysis showed TPC6A physically interacted with WWOX in resting cells. PreIP=~40 *μ*g of protein in the input loading. (**e**) COS7 cells were co-transfected with EYFP-TPC6AΔ and ECFP-WWOX or indicated domains of WWOX. FRET microscopy revealed that TPC6AΔ binds WWOX to its C-terminal D3 tail (see increased FRETc). The relative binding strength is shown in a color scale, where the highest binding energy is indicated in red. (**f**) Designated WWOX domains for interacting with TPC6AΔ are determined by FRET microscopy. Ten cells from each experimental set were analyzed by Image Pro Plus 6.1 (mean±S.D.; **P<0.05, Student’s *t*-test).

Next, we investigated whether WWOX physically binds TPC6A and affects each other’s function. We have determined that downregulation of WWOX leads to TPC6A aggregation both *in vitro* and *in vivo.*^1^ Transiently overexpressed EGFP-WWOX exhibited perinuclear distribution in the cytoplasm and the mitochondria of COS7 fibroblasts and many other types of cells.^15,35^ Co-expression of ECFP-TPC6A (or ECFP-TPC6AΔ) and EYFP-WWOX in COS7 cells resulted in co-localization at the perinuclear and nuclear areas (Figures 3a and b). Interestingly, co-expression of ectopic WWOX and TPC6AΔ significantly increased accumulation of WWOX in the nuclei (78±3.2% nuclear localization) compared with the expression of ectopic WWOX alone (42±12% nuclear localization; Figures 3a and b). Similar results were observed for the wild-type TPC6A. The observations suggest that TPC6A or TPC6AΔ binds WWOX and both proteins co-translocate to the nucleus. Alternatively, TPC6A may act as a carrier for WWOX to relocate to the nucleus.

We utilized co-IP and time-lapse FRET microscopy to determine the binding of WWOX with TPC6A and map their binding domains. Embryonic kidney fibroblast HEK293 cells were grown in a 10-cm Petri dish to almost 100% confluence, and then processed for immunoprecipitation using a specific antibody against TPC6A. By western blotting, endogenous TPC6A was shown to physically interact with WWOX (Figures 3c and d). Similarly, by using WWOX antibody, the WWOX/TPC6A complex was also observed (Figures 3c and d). Non-immune sera did not precipitate WWOX or TPC6A (data not shown). Similar results were observed using other cell lines such as COS7, SK-N-SH neuroblastoma, MCF7 breast cancer and L929 fibroblasts.

Next, COS7 cells were transiently overexpressed with ECFP-TPC6AΔ and EYFP-WWOX. FRET microscopy was carried out and the protein/protein binding energy was calculated as FRET concentration (FRETc).^7,22,28,34^ TPC6AΔ physically interacted with WWOX (with a significantly higher FRETc) compared with the ECFP/EYFP interaction (Figures 3e and f). To map the binding domain(s) in WWOX, the C-terminal SDR domain/D3 tail (aa #120–414), rather than the WW domains (aa #1–95), bound TPC6AΔ. To further narrow down the binding region, we determined that the C-terminal D3 tail physically bound TPC6AΔ (Figures 3e and f). The C-terminal D3 tail induces apoptosis when transiently overexpressed.^34,37^

### Transiently overexpressed TPC6AΔ induces apoptosis and counteracts the function of WWOX in activating promoter governed by NF-*κ*B

**Figure 4:**
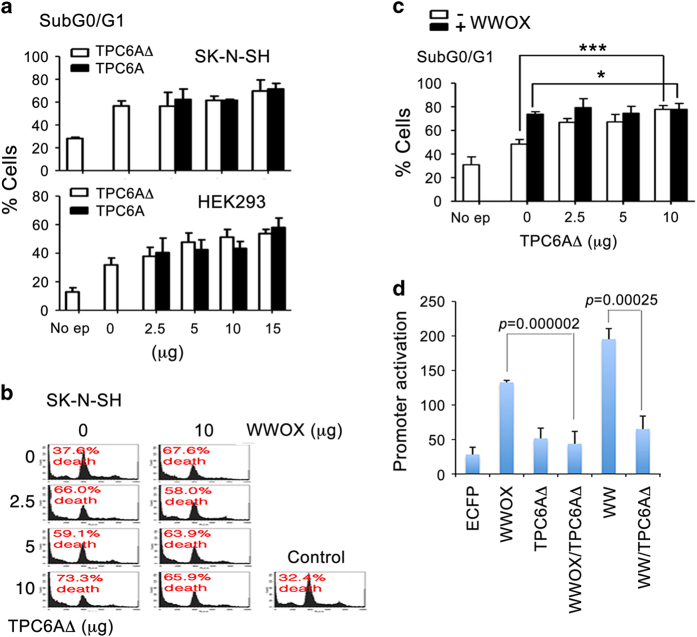
TPC6A fails to induce apoptosis synergistically with WWOX. (**a**) Transiently overexpressed TPC6AΔ and TPC6A were equally potent in inducing apoptosis of SK-N-SH and HEK293 cells in a dose-dependent manner. No ep indicates no electroporation. (**b** and **c**) SK-N-SH cells were transiently overexpressed with EYFP-TPC6AΔ and EYFP-WWOX, and both proteins failed to induce apoptosis in an additive manner (mean±S.D.; *n*=3; *P>0.1, ***P<0.005; Student’s *t*-test). (**d**) An assay for NF-*κ*B-governed promoter activation was carried out (Li *et al.*^22^). WWOX and its WW domain-induced promoter activation were significantly blocked by TPC6AΔ (*n*=10).

We examined the functional relationship between WWOX and TPC6AΔ. Transiently overexpressed TPC6A and TPC6AΔ induced apoptosis of neuroblastoma SK-N-SH cell and HEK293 fibroblasts (Figure 4a), as well as many types of cells (data not shown). There were no significant differences regarding the efficacy of TPC6A and TPC6AΔ in inducing apoptosis. Transiently overexpressed WWOX induces apoptosis via the mitochondrial pathway.^10,15,35,38,39^ Interestingly, TPC6A and WWOX nullify each other’s function in causing apoptosis. For example, when transiently overexpressed, both WWOX and TPC6A induced apoptosis (Figures 4b and c). However, in combination, there were no additive effects (Figures 4b and c), suggesting that both proteins counteract each other’s function. To further test this notion, transiently overexpressed TPC6AΔ abolished the function of WWOX in activating NF-*κ*B promoter (Figure 4d). We have previously determined that the WW domain of WWOX strongly activates the promoter governed by NF-*κ*B for increasing cell survival.^22^

### WWOX prevents TPC6AΔ aggregation

**Figure 5:**
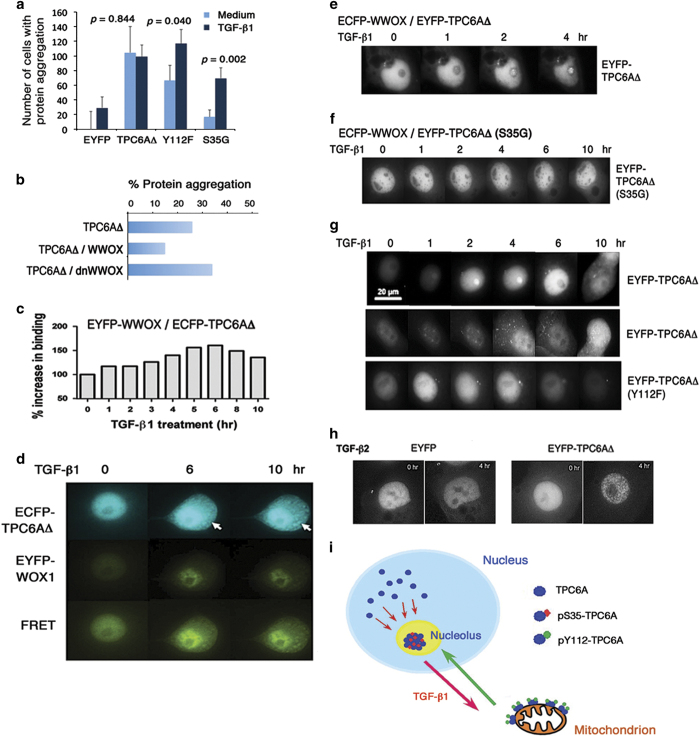
WWOX binds and blocks TPC6AΔ aggregation, and TGF-*β*1 dissociates TPC6AΔ from WWOX for leading to aggregation. (**a**) COS7 cells were co-transfected with expression constructs for EYFP, EYFP-TPC6AΔ, EYFP-TPC6AΔ(S35G) and/or EYFP-TPC6AΔ(Y112F), followed by culturing for 24 h and then treating with or without TGF-*β*1 (5 ng/ml) for another 24 h. The extent of protein aggregation was measured. TGF-*β*1-induced protein aggregation was statistically analyzed (control *versus* treated samples; ~100 cells analyzed; *n*=3). (**b**) COS7 cells were transfected with ECFP-WWOX and/or EGFP-TPC6AΔ by electroporation. Forty-eight hours later, ectopic TPC6AΔ underwent aggregation, and WWOX suppressed the aggregation by ~50%. Dominant negative WWOX (dn-WWOX) failed to block the aggregation of TPC6AΔ. Approximately 300 green fluorescent cells in total were counted, and the average is shown. (**c** and **d**) COS7 cells were transiently expressed with EYFP-WWOX and ECFP-TPC6AΔ. After 24 h in culture, cells were treated with TGF-*β*1 (5 ng/ml) and time-lapse FRET microscopy was performed. TGF-*β*1 increased the binding of WWOX with TPC6AΔ initially in the nucleus for 6 h, followed by reduction in the binding and increased TPC6AΔ aggregation in the cytoplasm. Also, see Supplementary Videos 1,2,3. (**e** and **f**) COS7 cells were transiently expressed with ECFP-WWOX and EYFP-TPC6AΔ or EYFP-TPC6AΔ(S35G). Upon exposure to TGF-*β*1 (5 ng/ml), ectopic WWOX and TPC6AΔ became aggregated in the nucleolus, whereas no aggregation was shown for WWOX and TPC6AΔ(S35G). The cell started undergoing apoptosis at hour 15 (see Supplementary Video 6). (**g**) Similarly, aggregation of EYFP-TPC6AΔ in the nucleolus (top panel) or cytoplasm (middle panel) was observed upon exposure of COS7 cells to TGF-*β*1 (5 ng/ml). Alteration of Tyr112 to Phe112 resulted in the failure of relocation of the mutant protein to the cytoplasm. (**h**) Under similar conditions, TGF-*β*2 (5 ng/ml) induced aggregation of EYFP-TPC6AΔ in the nucleus in 4 h. (**i**) In summary, two-way endogenous TPC6A shuttling is illustrated. In response to TGF-*β*1, nuclear TPC6A undergoes Ser35 phosphorylation for entering the nucleoli and then relocates out to the mitochondria as a dimer, which probably requires phosphorylation at Tyr112. Again, TPC6A migrates back to the nucleoli. Ectopic TPC6AΔ, tagged with EGFP or EYFP, undergoes one-way trafficking from the nuclei to the mitochondria only. This is due to TPC6AΔ protein aggregation in the mitochondria.

We have recently determined that Ser35 phosphorylation is essential for TPC6AΔ aggregation, whereas Tyr112 is less effective.^1^ When COS7 cells were transfected with the expression construct for EYFP, EYFP-TPC6AΔ, EYFP-TPC6AΔ(S35G) or EYFP-TPC6AΔ(Y112F), followed by culturing for 24 h and then treating with TGF-*β*1 for another 24 h. As expected, the S35G mutant failed to undergo aggregation, and the Y112F mutant had a reduced aggregation (Figure 5a). Interestingly, TGF-*β*1 significantly increased the aggregation of the S35G mutant (Figure 5a).

When WWOX expression is knocked down by small interfering RNA, aggregation of TPC6AΔ and TIAF1 occurs.^1^ The observation supports the notion that the D3 tail of WWOX binds and blocks TPC6AΔ aggregation. By protein aggregation assay, transiently overexpressed TPC6AΔ alone became aggregated in COS7 fibroblasts (Figure 5b). In co-expression with WWOX, TPC6AΔ aggregation was suppressed by ~50%. Dominant negative WWOX (dn-WWOX), which blocks the apoptotic function of WWOX and p53,^38,39^ failed to block the aggregation of TPC6AΔ (Figure 5b). Compared with TPC6AΔ, wild-type TPC6A could not undergo aggregation effectively.^1^ WWOX had no apparent effect on the aggregation of wild-type TPC6A (data not shown). Together, these observations suggest that the D3 tail of WWOX binds TPC6AΔ, and this binding blocks the aggregation of TPC6AΔ.

### TGF-*β*1 induces dissociation of the WWOX/TPC6AΔ complex for leading to TPC6AΔ aggregation and cell death

When COS7 cells were transiently overexpressed with ECFP-TPC6AΔ and EYFP-WWOX (or ECFP and EYFP) and exposed to TGF-*β*1, there was an initial increase in the binding of TPC6AΔ with WWOX in the nucleus, followed by reduction, as determined by time-lapse FRET microscopy (Figures 5c and d and Supplementary Videos 1,2,3). That is, the binding strength of TPC6AΔ and WWOX reached a maximal extent in 6 h in the nucleus. Later, a portion of both proteins relocated to the cytoplasm and underwent aggregation, as revealed by the positive FRET signal in the cytoplasm (see arrows in Figure 5d and Supplementary Videos 1,2,3). In most cases, the cells underwent apoptosis during a prolonged period of treatment with TGF-*β*1 for 24–48 h. No protein aggregation was observed in control cells overexpressing ECFP and EYFP (data not shown). Collectively, during a prolonged stimulation, TGF-*β*1 induces dissociation of the WWOX/TPC6AΔ complex that leads to TPC6AΔ aggregation and eventual cell death.

### Ser35 phosphorylation is essential for relocation of TPC6AΔ to the nucleolus and pTyr112 needed for relocation to the cytoplasm

Alternatively, WWOX was tagged with ECFP and TPC6AΔ with EYFP. EYFP-TPC6AΔ relocated to the nucleolus and became self-aggregated in 2 h upon treatment of COS7 cells with TGF-*β*1 (Figure 5e and Supplementary Video 4). This result is in agreement with the observation that TGF-*β*1 induced shuttling of endogenous TPC6A to the nucleolus (Figure 2). Without exposure to TGF-*β*1, there was no translocation of EYFP-TPC6AΔ to the nucleolus (Supplementary Video 5). Alteration of Ser35 to Gly35 abolished TGF-*β*1-induced relocation of EYFP-TPC6AΔ(S35G) to the nucleolus (Figures 5a and f and Supplementary Video 6). EYFP-TPC6AΔ(S35G) did not undergo aggregation when transiently overexpressed (Figure 5f). Notably, when the strength of binding of ECFP-WWOX with EYFP-TPC6AΔ(S35G) reached maximally at hour 15, the cell underwent apoptosis probably due to the aggregation of WWOX with TPC6AΔ(S35G) (Supplementary Video 6). The EYFP-TPC6AΔ(Y112F) mutant failed to relocate to the cytoplasm (bottom in Figure 5g).

Without ectopic WWOX, aggregation of TPC6AΔ occurred effectively. When COS7 cells were overexpressed with EYFP-TPC6AΔ only, TGF-*β*1 induced relocation of TPC6AΔ to the nucleolus, followed by migrating out to the cytoplasm in less than 6 h (two representative data at top and middle rows from the same video; Figure 5g and Supplementary Video 7). Again, by time-lapse microscopy, we showed TGF-*β*1 induced nuclear EYFP-TPC6AΔ relocation to the mitochondria (Supplementary Video 8). Mitochondria were stained with MitoTracker Red. We also obtained similar results by treating cells with TGF-*β*2, which showed the induction of EYFP-TPC6AΔ aggregation in the nuclei and cytoplasm in 2 h (Figure 5h and Supplementary Video 9).

Taken together, there is a two-way shuttling for endogenous TPC6A and TPC6AΔ (Figures 2 and 5i). In response to TGF-*β*1, nuclear TPC6A undergoes Ser35 phosphorylation for entering the nucleoli, and then relocates out to the mitochondria as a dimer with a likely phosphorylation at Tyr112 (Figure 5i). Again, TPC6A travels back to the nucleoli. However, transiently overexpressed TPC6AΔ did not relocate back to the nuclei and became accumulated in the mitochondria.

### Induction of mitochondrial apoptosis causes nuclear TPC6AΔ to relocate to the cytoplasm

**Figure 6:**
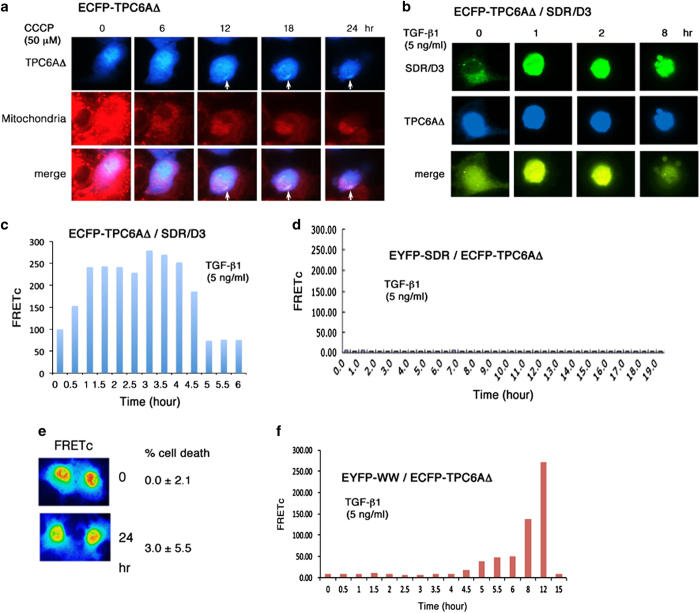
TPC6AΔ and D3 act synergistically in TGF-*β*1-mediated apoptosis, whereas the WW domain counteracts the event. (**a**) To induce mitochondrial apoptosis, ECFP-TPC6AΔ expressing COS7 cells were treated with CCCP. Time-lapse microscopy was carried out to chase the loss of mitochondrial membrane permeability (stained with MitoTracker Red) and relocation of ECFP-TPC6AΔ to the cytoplasm, along with aggregation (see white arrows), with time (also see Supplementary Videos 10 and 11). (**b** and **c**) COS7 cells, transiently overexpressing with TPC6AΔ and SDR/D3, were treated with TGF-*β*1 for time-lapse FRET microscopy (also see Supplementary Videos 12,13,14,15). TGF-*β*1 increased the binding of TPC6AΔ with SDR/D3 with time followed by reduction and occurrence of cell death. During cell death, increased secretion of exosome-like particles to the extracellular space is shown (Supplementary Video 14). (**d**) TGF-*β*1 failed to induce the binding of TPC6AΔ with SDR domain and no cell death occurred (data not shown). (**e** and **f**) Interestingly, TGF-*β*1 increased the binding of TPC6AΔ with WW domain during exposure for 8–12 h, whereas no cell death occurred.

In comparison, CCCP (carbonyl cyanide m-chlorophenyl hydrazone), an inducer of mitochondrial apoptosis, caused relocation of TPC6AΔ from the nuclei to the mitochondria. When COS7 cells were transiently overexpressed with TPC6AΔ and then treated with CCCP, CCCP rapidly induced loss of mitochondrial membrane permeability in less than 1 h (loss of red fluorescence), as determined by time-lapse microscopy. TPC6AΔ started to relocate to the cytoplasm at hour 3 and became aggregated at hour 6 (see arrows in Figure 6a and Supplementary Videos 8 and 9). Post aggregation, the cells underwent apoptosis (Supplementary Videos 8 and 9). In negative controls, EGFP alone did not relocate to the mitochondria (data not shown). Similar results were observed when cells were exposed to UV irradiation. Relocation of TPC6AΔ from the nuclei to the mitochondria was observed (data not shown).

### TGF-*β*1 regulates the complex formation of TPC6AΔ with WWOX, and apoptosis occurs when the complex dissociates

We continued to examine how WWOX regulates TPC6AΔ aggregation and apoptosis. COS7 cells were transiently overexpressed with TPC6AΔ and SDR/D3. Upon exposure to TGF-*β*1, there was a rapid increased binding between TPC6AΔ and SDR/D3 in 30 min in COS7 cells, and the binding lasted for 4 h followed by dissociation. When TPC6AΔ dissociated from SDR/D3, apoptosis occurred (see apoptotic bodies in Figures 6b and c and Supplementary Videos 10,11,12,13). During cell death, increased secretion of exosome-like particles to the extracellular space is shown (Supplementary Video 12). In an appropriate control, TGF-*β*1 did not induce the binding of TPC6AΔ with SDR only (Figure 6d), and no cell death occurred. We observed the release of exosome-like particles to the extracellular matrix during phorbol ester-induced cell death,^28^ as well as in UV/cold shock-induced bubbling cell death from the nucleus.^40^ However, the functional properties of these exosome-like particles remain to be established.

Without stimulation, TPC6AΔ did not bind the WW domain (Figures 3e and f). When COS7 cells were transiently overexpressed with TPC6AΔ and WW domain only, TGF-*β*1 did not cause cell death (Figures 6e and f). Notably, upon stimulation with TGF-*β*1 for 6–8 h, there was an increased binding of TPC6AΔ with WW (Figures 6e and f). This binding and then dissociation did not render cell death. The observations imply that the WW domain of WWOX may provide a rescue mission to block apoptosis.

### TPC6A plaques interact with TIAF1 aggregates *in vivo*

**Figure 7:**
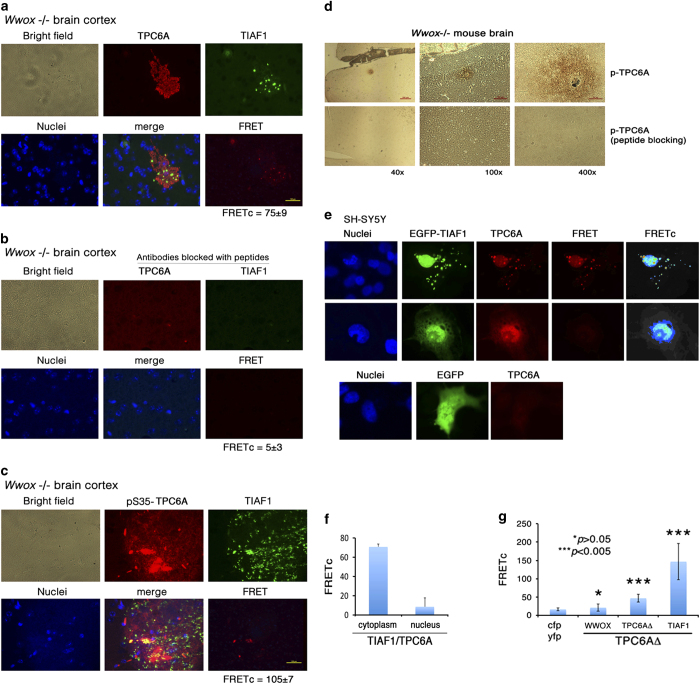
Binding of TPC6AΔ with TIAF1 *in vivo*. (**a**) By antibody-FRET, we determined the binding of TPC6A plaques (red) with TIAF1 aggregates (green) in the brain cortex of *Wwox*−/− mice. The binding affinity is expressed as FRETc (*n*=6; mean±S.D.). (**b**) In negative controls, no binding was observed. No primary antibodies were used. (**c**) Ser35-phosphorylated TPC6A also binds TIAF1. (**d**) By immunohistochemistry, we showed the pS35-TPC6A plaque in the brain cortex of *Wwox*−/− mice. (**e** and **f**) Transient overexpression of EGFP-TIAF1 in SH-SY5Y cells resulted in upregulation of TPC6A expression and their binding in the cytoplasm, but not in the nucleus (*n*=15, mean±S.D.). (**g**) COS7 cells were transiently expressed with ECFP-TPC6AΔ and EYFP-WWOX, EYFP-TPC6AΔ or EYFP-TIAF1. By FRET microscopy, we showed TIAF1 bound strongly with TPC6AΔ, but the binding was weak in other combinations (*n*=15, mean±S.D.). No binding was shown for ECFP and EYFP only.

By fluorescent immunostaining, we showed the presence of TPC6A plaques with Ser35 phosphorylation (red) and TIAF1 aggregates (green) in the brain cortex of *Wwox*−/− mice (Figures 7a–c). The observation suggests that formation of TPC6A plaques occurs first, followed by TIAF1 aggregation. This assumption has been approved by using siRNA to knock down WWOX, TPC6A and TIAF1, respectively.^1^ Presence of TPC6A and TIAF1 aggregates or plaques was also shown in the extracellular matrix in postmortem human AD hippocampi (Supplementary Figure S2). By antibody-FRET analysis, we showed the binding of TPC6A with TIAF1 (Figures 7a and c). In negative controls, there was no binding (Figure 7b). In addition, we showed the p-TPC6A plaques in the brain cortex of *Wwox*−/− mice by immunohistochemistry (Figure 7d).

### TIAF1 reciprocally increases TPC6A expression

Intriguingly, transient overexpression of EGFP-TIAF1 in SH-SY5Y cells significantly raised the expression of endogenous TPC6A (Figure 7e). Binding of EGFP-TIAF1 with endogenous wild-type TPC6A occurred mainly in the cytoplasm, but not in the nucleus (Figure 7f). To further determine the relationship between WWOX, TPC6AΔ and TIAF1, we showed that ectopic EYFP-TIAF1 bound strongly with ECFP-TPC6AΔ, as determined by FRET microscopy (Figure 7g). However, binding of ECFP-TPC6AΔ with EYFP-WWOX or EYFP-TPC6AΔ was relatively weak (Figure 7g). The results *in vitro* are in agreement with the observations *in vivo* (Figures 7a and c).

## Discussion

In this study, we have discovered for the first time that TGF-*β*1 induces two-way shuttling of TPC6A and TPC6AΔ in between nucleoli and mitochondria. The nucleolus–mitochondrion shuttling appears to be a rare event, although many proteins undergo one-way nucelocytoplasmic shuttling.^41^ One study reported that SenP5 translocates from the nucleoli to the mitochondria to modulate DRP1-dependent fission during mitosis.^42^ In yeast, TPC6A participates in tethering of the trafficking protein particle complex to the cis-Golgi membrane.^2^ In contrast, mammalian TPC6A is mainly localized in the nuclei and the perinuclear regions in mammalian cells. Although endogenous TPC6A and TPC6AΔ may polymerize in the cell compartments, the polymerized proteins do not induce cell death. Presumably, a novel cellular system depolymerizes the aggregated proteins before relocation. Site-directed mutagenesis showed that phosphorylation of Ser35 and Tyr216 is needed for TPC6A to relocate to the nucleolus and mitochondria, respectively. Endogenous WWOX binds TPC6A and reduces the time of round-trip shuttling by 50%. The underlying mechanism of this regard is unknown. Nonetheless, TPC6A nullifies WWOX-mediated activation of the responsive element governed by NF-*κ*B. The observations suggest that endogenous activated WWOX provides a pro-survival signal by activating NF-*κ*B under the stress of neurodegeneration, and its action can be overridden by TPC6A and other transcription factors.^22^

TPC6AΔ binds the D3 tail of WWOX. TGF-*β*1 dissociates the binding, which leads to the aggregation of TPC6AΔ in the mitochondria for causing apoptosis. Indeed, WWOX has an intramolecular folding via the binding of the N-terminal WW domain with the C-terminal SDR domain (Chen *et al.*, unpublished). TGF-*β*1 activates WWOX to phosphorylate at Tyr33.^34^ The activated form probably has an unfolded WW-SDR binding, thus allowing the exposure of WW domain for interacting with TPC6AΔ and preventing the aggregation-induced cell death. That is, during TPC6AΔ-induced cell death in response to TGF-*β*1, WWOX provides the first line of protection against apoptosis via D3 binding with TPC6AΔ.

Functional TGF-*β*/Smad signaling is believed to protect against the progression of AD.^43^ However, aberrant TGF-*β* signaling caused by interaction of Smads with protein tangles may facilitate AD progression.^44^ TGF-*β*1 expression is significantly increased in a transgenic mouse model of familial Alzheimer's disease and causes neuronal apoptosis.^32^ In stark contrast, blocking TGF-*β*-Smad2/3 innate immune signaling mitigates the Alzheimer-like pathology.^45^ We have shown that TGF-*β* induces TIAF1 self-aggregation via type II receptor-independent signaling that leads to the generation of amyloid *β* plaques in Alzheimer's disease.^7^ That is, TGF-*β* binds membrane hyaluronidase Hyal-2 that recruits WWOX and Smad4. In traumatic brain death, the TGF-*β*/Hyal-2/WWOX/Smad4 signaling can lead to neuronal death (Chen *et al.*, unpublished). By filter retardation assay, we have recently determined that protein aggregates of TPC6A and TIAF1 are present in the hippocampus of normal middle-aged individuals, and the TPC6A/TIAF1 complexes possess increasing amounts of A*β* in AD patients.^1^ The aggregated TPC6AΔ induces TIAF1 aggregation and activates caspases for causing APP degradation and A*β* generation. Caspase activation has been shown in the AD brains.^46^

Upon stimulation with TGF-*β*, endogenous TPC6A relocates to the nucleus and then nucleolus, and finally migrates out of the nucleolus to the cytoplasm or mitochondria. The role of TPC6A in the nuclei is unknown. A likely scenario is that TPC6A may carry RNAs or proteins out of the nucleoli, which are needed for mitochondria. A leucine zipper motif is predicted near the C-terminal of TPC6A, suggesting that TPC6A might be a DNA-binding protein. We also predicted two possible phosphorylation sites in TPC6AΔ, Ser35 and Tyr112. Alteration of Ser35 to Gly abolishes TGF-*β*-mediated aggregation of TPC6AΔ. The Ser35 phosphorylation has been verified by our produced phospho-antibody.^1^ However, alteration of Tyr112 to Phe does not reduce TPC6AΔ aggregation in the presence or absence of TGF-*β*. Importantly, overexpressed wild-type TPC6A may undergo aggregation, but fails to activates caspases. The binding affinity of TPC6AΔ with wild-type TPC6A is weak, and the binding does not induce caspase activation (data not shown).

Finally, the relationship among WWOX, TIAF1 and TPC6A is being determined. Knockdown of WWOX causes aggregation of TIAF1 and TPC6A.^1^ Interestingly, TPC6A knockdown induces TIAF1 aggregation, whereas TIAF1 knockdown does not induce TPC6A aggregation effectively. That is, the sequential cascade of aggregation is related to WWOX downregulation, which leads to the aggregation of TPC6A followed by TIAF1. The TPC6A and TIAF1 aggregates can be found in the mitochondria of degenerative neurons. ^1^ Most recently, we determined that overexpressed TIAF1 exhibits as aggregates together with Smad4 and A*β* in the cancer stroma and peritumor capsules of solid tumors.^30^ Also, TIAF1/A*β* aggregates are shown on the interface between brain neural cells and the metastatic cancer cell mass. TIAF1 is upregulated in developing metastatic tumor, but may disappear in established metastatic tumors. Growing neuroblastoma cells on the extracellular matrices from other cancer cell types induces the production of aggregated TIAF1 and A*β*.^30^ We have shown that TIAF1, p53 and WWOX act synergistically in suppressing anchorage-independent growth, blocking cell migration and causing apoptosis.^30,36^

## Materials and Methods

### Cell lines

Cell lines used in these studies were monkey kidney COS7 fibroblasts, human embryonic kidney HEK293 fibroblasts, human squamous cell carcinoma SCC-9 cells, human basal cell carcinoma BCC cells, human colon cancer HCT116 cells, human neuroblastoma SK-N-SH, mouse melanoma B16F10 cells and primary rat glial cells. The cell lines were grown and maintained according to the instructions of ATCC (American Type Culture Collection, Manassas, VA, USA).

### cDNA expression constructs, mutant clones, transient gene expression and antibodies

Human TPC6A and TPC6AΔ were constructed in pECFP-C1, pEGFP-C1 and pEYFP-C1 (Clontech, Mountain View, CA, USA), respectively, as described.^1^ Clones included the full-length TPC6A, TPC6AΔ and mutant TPC6AΔ(S35G) and TPC6AΔ(Y112F). Expression constructs of full-length and truncated WWOX, including WW domain, SDR domain, D3 tail and SDR/D3 region, were made as previously described.^7,15,38,39^ Transient gene expression of indicated cDNA expression constructs by electroporation was performed as described.^7,15,38,39^ Specific antibodies against WWOX, pY33-WWOX, TIAF1, TPC6A, TPC6AΔ and phosphorylation at pSer35 were made as described.^7,8,15,20,34,38,39^ Antibody against *α*-tubulin was from Sigma (St. Louis, MO, USA).

### Co-IP and western blotting

Co-IP and western blotting analyses were performed as described previously.^7,15,38,39^ Where indicated, cells were cultured in 100-mm Petri dishes to 100% confluence, followed by harvesting. Cell lysates were prepared and processed for co-IP using specific antibodies, respectively, against TPC6A and WWOX.

### Time-lapse Förster/FRET microscopy

To analyze protein physical interactions, FRET analysis was performed.^7,22,28,34^ Briefly, COS7 cells were transfected with TPC6AΔ-pEYFPC1 and WWOX-pECFPC1 or indicated WWOX domain constructs with ECFP tag. After culturing for 24–48 h, cells were subjected to time-lapse microscopy in the presence or absence of TGF-*β*1 (5 ng/ml; PeproTech, Rocky Hill, NJ, USA). Cells were then stimulated with an excitation wavelength 440 nm, and the FRET signals were detected at an emission wavelength 535 nm by time-lapse fluorescence microscopy. Background fluorescence of the FRET signal was corrected, and FRET concentration (FRETc) was calculated using Youvan’s method [Image Pro 6.1 (Media Cybernetics, Rockville, MD, USA)]: FRETc=(fret−bk[fret])−cf[don]×(don−bk[don])−cf[acc]×(acc−bk[acc]); where FRETc=FRET concentration, fret=FRET imaging, bk=background, cf=correction factor, don=donor image, acc=acceptor image.

### Cell cycle analysis

Cell cycle analysis was performed.^15,38^ Briefly, the cells were transfected with TPC6A-pEYFP-C1, TPC6AΔ-pEYFP-C1 and/or WWOX-pEYFP-C1. After culturing for 24–48 h, cells were harvested by centrifugation at 2000 r.p.m., gently washed once with PBS and finally fixed with 75% ethanol. Fixed cells were precipitated by centrifugation, washed once by PBS and then stained with propidium iodide (PI) solution (2 *μ*g/ml PI, 10 *μ*g/ml RNase A in PBS) for 30 min at room temperature. Flow cytometry/FACS (Becton Dickinson, BD, Franklin Lakes, NJ, USA) analysis was carried out to determine DNA contents. Three independent experiments were performed, and Student’s *t*-test was used to analyze data among the controls and experiments.

## Abbreviations

AD: Alzheimer’s disease
A*β*: *β*-amyloid
APP: amyloid precursor protein
BACE: APP *β*-secretase
FRET: Förster (fluorescence) resonance energy transfer
FTD: frontotemporal dementia
HD: Huntington disease
PMA: phorbol 12-myristate 13-acetate
NFT: neurofibrillary tangles
TGF-*β*: transforming growth factor beta
TIAF1: TGF-*β*-induced antiapoptotic factor 1
TRAPP: trafficking protein particle complex
TRAPPC6A (TPC6A): TRAPP complex 6A
wtTPC6A: wild-type TPC6A
TPC6AΔ: an N-terminal internal deletion isoform of wtTPC6A
WWOX or WOX1: WW domain-containing oxidoreductase.
