# Supplementary material for: WWOX dysfunction induces sequential aggregation of TRAPPC6AΔ, TIAF1, tau and amyloid β, and causes apoptosis
Source: Cell Death Discov. 2015 Aug 3;1:15003–. doi: 10.1038/cddiscovery.2015.3 (PMC4981022; doi:10.1038/cddiscovery.2015.3)
Supplement: Supplementary Figures [file cddiscovery20153-s1.doc]

**SUPPLEMENTAL MATERIALS**

**Figure S1. Colocalization of TIAF1 with TPC6A in postmortem AD hippocampi.**

(**A**) Human postmortem hippocampal tissue sections were were pre-stained with Bielschowski stain (containing silver), followed by staining with specific antibodies against TIAF1 (green) and p-TPC6A (for S35 phosphorylation; red), respectively, and then with secondary antibodies. Nuclei were stained with DAPI (400x magnification).

(**B**) In negative controls, no primary antibodies were used in the immunostaining.


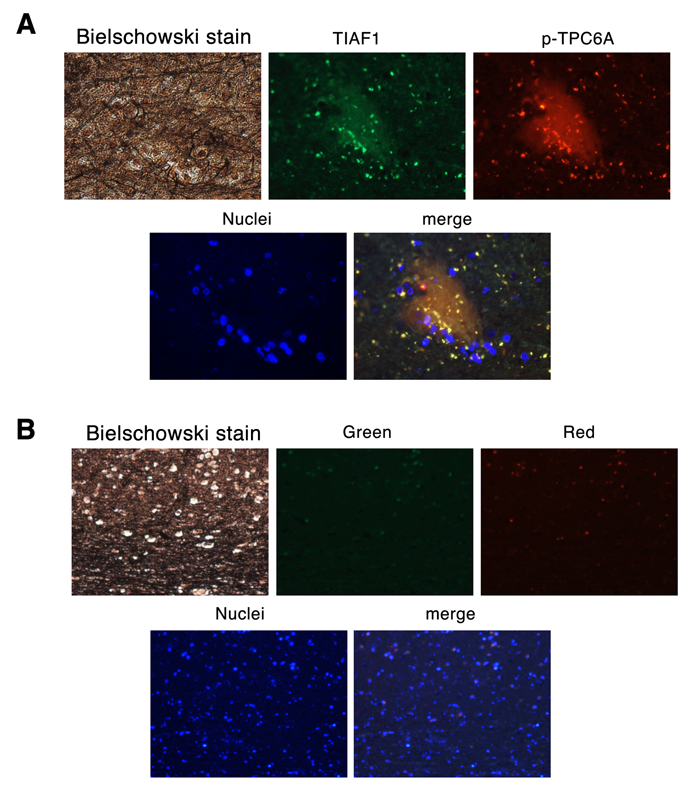


**Figure S2. TPC6A and TIAF1 aggregates in the extracellular matrix in postmortem human AD hippocampi.** (**A,B**) Archival AD hippocampal tissue sections were processed with Bielschowsky stain (right column), followed by staining with TPC6A antiserum and then with fluorescent secondary antibody. Bielschowsky stain abolished autofluorescence in the tissue sections (Lee et al., 2010). TPC6A aggregates (red) are shown in the plaques of hippocampus. Residual nuclei or DNA (stained with DAPI) in the aggregates indicates the presence of dead neurons. Scale bars for 200X and 400X magnifications are 50 μm and 20 μm, respectively. (**C**) In the negative controls, no signals are shown using fluorescent secondary antibody, followed by DAPI. Scale bar: 20 μm. (**D**) TPC6A (green) and WOX1 or WWOX (red) colocalize in normal neurons (yellow; 400x magnification). (**E,F**) TPC6A aggregates, without colocalization with WOX1 or amyloid beta (Aβ), are present in extracellular space in the human AD hippocampus (400x magnification). (**G-I**) TIAF1 and TPC6A aggregates colocalize in the plaque of AD hippocampus. Scale bar: 20 μm.

**
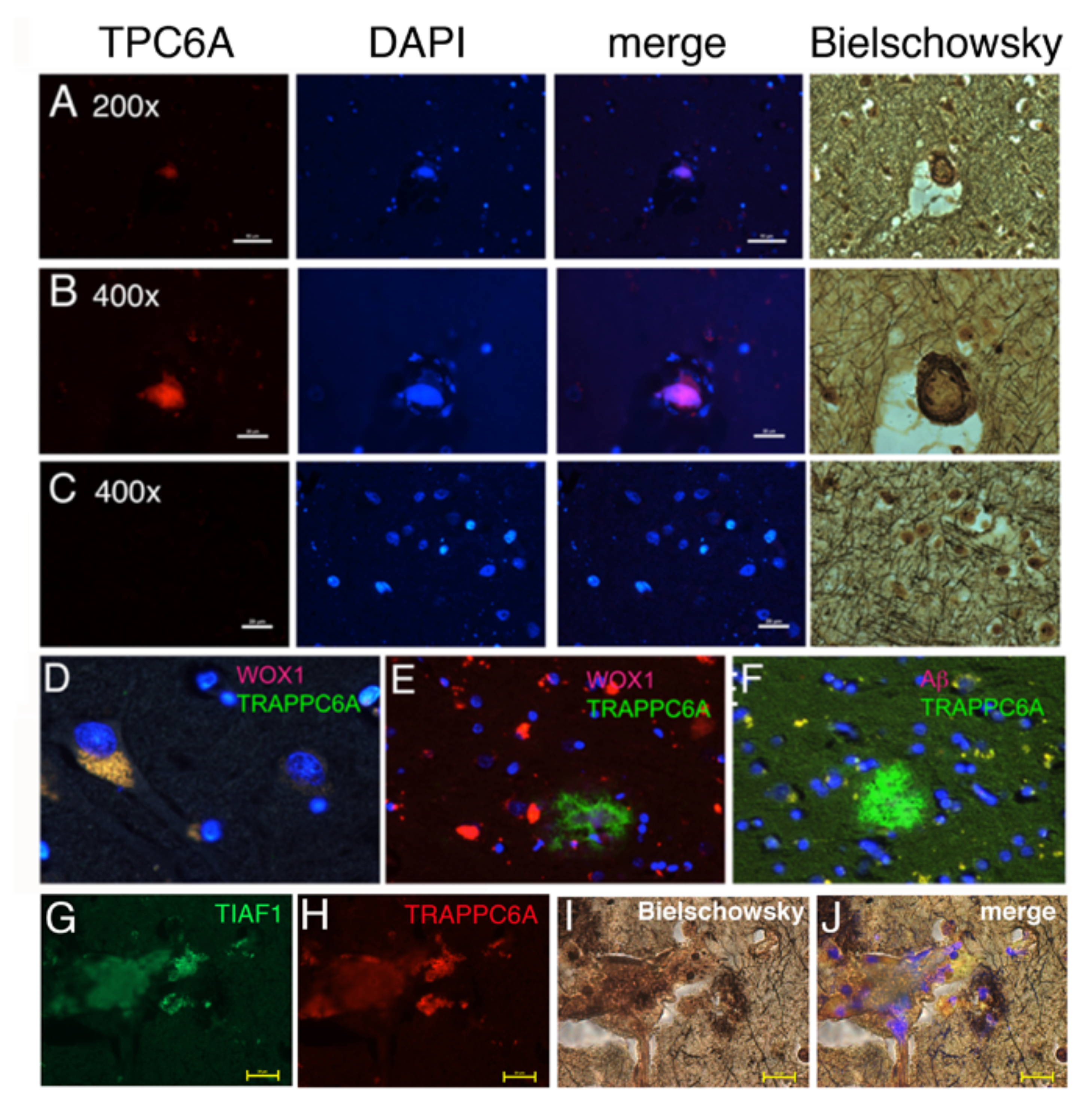
**
